# MicroRNA-142 Reduces Monoamine Oxidase A Expression and Activity in Neuronal Cells by Downregulating SIRT1

**DOI:** 10.1371/journal.pone.0079579

**Published:** 2013-11-11

**Authors:** Amrita Datta Chaudhuri, Sowmya V. Yelamanchili, Howard S. Fox

**Affiliations:** Department of Pharmacology and Experimental Neuroscience, University of Nebraska Medical Center, Omaha, Nebraska, United States of America; University of Missouri-Kansas City, United States of America

## Abstract

Aberrant expression of microRNAs (miRs) has been implicated in the pathogenesis of several neurodegenerative disorders. In HIV-associated neurocognitive disorders (HAND), miR-142 was found to be upregulated in neurons and myeloid cells in the brain. We investigated the downstream effects of chronic miR-142 upregulation in neuronal cells by comparing gene expression in stable clones of the human neuroblastoma cell line BE(2)M17 expressing miR-142 to controls. Microarray analysis revealed that miR-142 expression led to a reduction in monoamine oxidase (MAO) A mRNA, which was validated by qRT-PCR. In addition to the mRNA, the MAOA protein level and enzyme activity were also reduced. Examination of primary human neurons revealed that miR-142 expression indeed resulted in a downregulation of MAOA protein level. Although MAOA is not a direct target of miR-142, SIRT1, a key transcriptional upregulator of MAOA is, thus miR-142 downregulation of MAOA expression is indirect. MiR-142 induced decrease in MAOA expression and activity may contribute to the changes in dopaminergic neurotransmission reported in HAND.

## Introduction

MicroRNAs (miRs) fine-tune gene expression at the post-transcriptional level thereby regulating various cellular processes. They bind to the 3′ untranslated region (UTR) of target mRNAs and recruit the RNA-induced silencing complex (RISC) to downregulate expression of the target. MiRs have emerged as important regulators of neuronal function, their altered expression contributing to neuronal dysfunction in diseases of the central nervous system (CNS) [1], [2], including HIV-associated neurocognitive disorders (HAND) [3]–[8]. Since HIV does not infect neurons, the neuronal pathology of HAND is secondary to CNS inflammation [9]. In recent years, implementation of combined antiretroviral therapy (cART) has resulted in lower plasma and CSF viral load and higher CD4+ cell counts in HIV infected patients [10]. Therefore, HIV-infected patients now live longer. However, this chronicity may further pre-dispose them to age-related cognitive impairment and the prevalence of HAND has increased despite implementation of cART [11]. One pathological manifestation of HIV infection that can lead to a severe form of HAND is HIV encephalitis (HIVE), where inflammatory cytokines and chemokines as well as HIV proteins cause changes in neuronal gene expression, leading to neuronal dysfunction and death [9], [12]. Elucidation of novel molecular mechanisms that contribute to the neuronal dysfunction in HIVE is necessary, as it will provide insights into pathogenesis of not only HAND, but also other degenerative diseases associated with CNS inflammation.

Alteration of the brain miR expression profile in HIVE and its non-human primate model (simian immunodeficiency virus encephalitis, SIVE) has been reported in previous studies [3]–[7]. Among the miRs that were found to be differentially expressed in the disease condition compared to uninfected control samples, miR-142 was upregulated both in the frontal cortex white matter in humans [7], as well as in the caudate nucleus and hippocampus in monkeys and caudate nucleus in humans [6]. In a previous study we showed that in the brain, miR-142 is upregulated within neurons and macrophage/microglia nodules in SIVE [8]. We also identified the NAD-dependent deacetylase Sirtuin1 (SIRT1) as a direct target for miR-142-5p, one of the two functional mature forms of miR-142 [8]. MiR-142 has been extensively studied in the hematopoietic cell lineage, where it regulates differentiation of T lymphocytes and myeloid cells [13]–[16]. In addition to SIVE, miR-142 expression in neurons has been reported following nerve crush injury [17] and cocaine treatment [18]. However, very little is known about downstream effects of chronic miR-142 upregulation in neuronal cells. In this context, *in vitro* miR-142 has been shown to target the transcripts of key neuronal genes encoding the D1 dopamine receptor (DRD1) [19] and the Clock-partner aryl hydrocarbon receptor nuclear translocator-like (ARNTL or BMAL1) [20]-[22], both of which play important roles in neuronal function. These studies, like most with miRs, were conducted after transient overexpression of miR-142.

The goal of the present study was to identify neuronal genes affected by chronic miR-142 upregulation and that may contribute to the neuronal pathology in HAND. We report that chronic overexpression of miR-142 in a neuronal cell line leads to downregulation of expression and activity of the neurotransmitter-metabolizing enzyme monoamine oxidase (MAO) type A. Decrease in MAOA protein level was also confirmed in primary human neurons that were transduced with miR-142. The MAOA 3′UTR does not have any binding sites for either miR-142-3p or -5p. Previously we identified SIRT1 as a direct target of miR-142; SIRT1 is downregulated in cell lines and neurons overexpressing miR-142 [8] and is known to induce MAOA expression [23]. Therefore, we postulate that miR-142 decreases MAOA expression by reducing SIRT1 protein level. Accordingly, overexpression of SIRT1 restored MAOA protein expression levels. Downregulation of SIRT1 by miR-142 therefore leads to the reduction in MAOA expression and activity, and may contribute to the changes in catecholaminergic neurotransmission in HAND.

## Materials and Methods

### Ethics Statement

For primary human neuron culture, fetal brain tissue was obtained in full compliance with the ethical guidelines of the NIH from the Birth Defects Laboratory, University of Washington, Seattle, WA, USA, where written informed consent from the next of kin was obtained for use of this sample in research under Institutional Review Board approval (#96-1826-A07). All research work was performed under Institutional Review Board approval (#009-00-FB) from the University of Nebraska Medical Center, Omaha, NE.

### Cell lines and primary neuron culture

BE(2)M17 neuroblastoma cells (obtained from the American Type Culture Collection (ATCC), Manassas, VA, cat. #CRL-2267), were cultured in Dulbecco's Modified Eagle Medium with F12 and Glutamax (Gibco, NY, USA) containing 10% fetal bovine serum, 500 U/mL penicillin and 500 µg/mL streptomycin (Gibco, NY, USA). All cell lines were maintained at 37°C in 95% air and 5% CO_2_.

For primary human neuron culture, fetal brain tissue was incubated with 0.25% trypsin for 30 minutes, neutralized with 10% fetal bovine serum and further dissociated by trituration. The resulting single-cell suspension was cultured on poly-D-lysine coated plates in Neurobasal medium supplemented with 0.5 mM l-glutamine, 500 U/mL penicillin and 500 µg/mL streptomycin and B27 supplement (Gibco, NY, USA). Neurons were cultured in vitro for 11 days before any experiment. To confirm purity of primary neuron culture, they were stained for MAP2 (1∶1500) (Sternberger Monoclonals Inc, MD, USA).

### Preparation of stable BE(2)M17 clones expressing miR-142 and miR-null

BE(2)M17 neuroblastoma cells were transfected either with pEP-miR-142 or a pEP-miR-null plasmid (Cell Biolabs, CA, USA) using Neuromag transfection reagent (Oz Biosciences, France) according to manufacturer's protocol. Seventy-two hours after transfection, the medium was changed to selection medium containing 4 µg/mL puromycin. Three independent miR-142 and miR-null clones were picked and miR-142 expression was confirmed by qRT-PCR using TaqMan microRNA assay (Applied Biosystems, CA, USA) for miR-142-3p and -5p, and U6 snRNA as endogenous control. Relative expression was calculated using efficiency corrected Ct method as described previously [24].

### Microarray analysis and mRNA qRT-PCR for BE(2)M17 clones

Gene array was performed using GeneChip Human Exon 1.0 ST Array (Affymetrix, CA, USA) by UNMC microarray core facility and the results were analyzed using Partek Genomics Suite (Partek Incorporated, MO, USA). The microarray data have been deposited in NCBI GEO, accession #GSE50133. Genes downregulated in the miR-142 clones with a p-value <0.001 were validated by qRT-PCR, which was performed using TaqMan 96-well array (Applied Biosystems, CA, USA) with GAPDH, HPRT1 and GUSB as endogenous controls. Relative expression was calculated using efficiency corrected Ct method as described previously [24].

### Western Blotting

Whole cell lysates were prepared using RIPA buffer (50 mM Tris-HCl pH 8, 150 mM NaCl, 1% NP40, 0.5% sodium deoxycholate, 0.1% SDS) and protein quantification was carried out using Pierce BCA protein assay (Thermo Scientific, IL, USA). Five to 15 µg of protein was loaded in each lane of NuPAGE 4–12% Bis-Tris gels (Invitrogen, CA, USA). Separated proteins were transferred onto nitrocellulose membranes using iBlot (Invitrogen, CA, USA). The membranes were blocked in SuperBlock (TBS) blocking buffer (Thermo Scientific, IL, USA) and then incubated overnight at 4°C with primary antibody. The following primary antibodies were used: rabbit polyclonal Sirtuin 1 (SIRT1) (1∶200), rabbit polyclonal Monoamine Oxidase A (MAOA) (1∶400) (Santa Cruz Biotechnology, CA, USA), rabbit polyclonal actin (1∶5000) (Sigma Aldrich, MO, USA). This was followed by incubation with secondary antibody; horseradish peroxidase conjugated anti-rabbit IgG (1∶20,000) (Thermo Scientific, IL, USA), for 1 hour at room temperature. Blots were developed using SuperSignal West Pico Chemiluminescent Substrate (Thermo Scientific, IL, USA), imaged and quantified using Carestream MI software.

### Monoamine oxidase (MAO) enzyme activity assay

Mitochondria were isolated from miR-142 and miR-null expressing BE(2)M17 clones using mitochondria isolation kit for cultured cells (MitoSciences, OR, USA). Total MAO, MAOA and MAOB enzyme activity was quantified using Amplex Red Monoamine Oxidase Assay Kit (Invitrogen, CA, USA). The enzyme activity was normalized to total protein content in each sample.

### Preparation of miR-142 expressing lentivirus and transduction of primary human neurons

Lentivirus expressing miR-142 was prepared as described previously [25]. First, the BamH1 restriction site present upstream of the pre-miR-142 sequence in the pEP-miR-142 expression vector was mutated to a PacI restriction site using the following PCR primers.

Forward: 5′-tcgattaattaaccttggggggat-3′

Reverse: 5′-tcgagctagcggagg-3′

The PCR product was run on a 1% agarose gel and was extracted from the band corresponding to the length of pre-miR-142 fragment (approximately 300 base pairs) using QIAquick gel extraction kit (Qiagen, Valencia, CA). The FUGW vector was digested with PacI and NheI to generate sticky ends corresponding to that of the pre-miR-142 fragment from the mutated pEP-miR-142 expression vector. The pre-miR-142 fragment was then ligated to the FUGW vector using T4 DNA ligase (Invitrogen, Carlsbad, CA). The ligated DNA was transformed into Stbl2 cells. Several colonies were picked and screened for the pre-miR-142 sequence using double digestion with PacI and NheI followed by separation and visualization on 1% agarose gel. DNA from colonies that were positive for pre-miR-142 sequence was sent to the UNMC DNA sequencing core facility to confirm the presence of pre-miR-142 sequence.

HEK293T cells were transfected with miR-142-FUGW, Δ8.9 and vesicular stomatitis virus G protein using XtremeGene HP transfection reagent (Roche Applied Science, IN, USA) according to manufacturer's protocol. Cell supernatant containing virions was collect 48 hour and 72 hour after transfection, and concentrated by ultracentrifugation at 25,000 rpm for 90 minutes. FUGW without precursor miR-142 insert was used as control. Lentivirus titer was determined using HIV p24 ELISA assay (Express Biotech International, MD, USA).

Primary human neurons were grown in vitro for 11 days and transduced with miR-142 or control lentivirus at a concentration of 5×10^6^ lentiviral particles/mL. Successful transduction was confirmed by visualizing GFP expression. Six days after transduction cells were collected for Western blotting and qRT-PCR.

### Transient transfection of BE(2)M17 clones with SIRT1

Flag-tagged SIRT1 plasmid was obtained from Addgene (Addgene, Cambridge, MA, plasmid 1791) [26]. Transfections were preformed with Neuromag transfection reagent according to the manufacturer's protocol. Cells were harvested 48 hours after transfection.

### Statistical Analysis

Statistical analysis was performed using Prism software (GraphPad, CA, USA) unless otherwise stated. Paired or unpaired Student's t-test, or one-way ANOVA followed by Bonferroni's multiple correction test was performed as applicable.

## Results

### Stable overexpression of miR-142 in BE(2)M17 cells reduces MAOA mRNA expression level

To identify miR-142-regulated genes relevant to neuronal pathophysiology, we created stable clones of BE(2)M17 neuroblastoma cells expressing miR-142 or the control plasmid, denoted miR-null. The BE(2)M17 cell line was chosen because it does not express any endogenous miR-142. Overexpression of miR-142-3p and -5p in the stable BE(2)M17 clones was confirmed by performing qRT-PCR for the respective mature miRs. RNA was extracted from three independent miR-142 clones and three miR-null clones, and microarray analysis was performed to determine the differences in gene expression. Given the large number of genes examined, we used p-value cut-off of p<0.001 and a fold change >2.5 to identify the top candidate genes. The expression of eight genes was altered between the miR-142- and miR-null-expressing clones using these criteria. qRT-PCR revealed that all except one, that was not detectable, were decreased in the miR-142 expressing cells. However, the decrease in expression of only MAOA and AKAP12 reached significance (Table 1). As MAOA is an important neurotransmitter-metabolizing enzyme, the decrease in expression of which may contribute to neuronal dysfunction, we chose to focus on MAOA for further studies.

**Table 1:** Summary of microarray and qRT-PCR analyses comparing differences in gene expression between stable BE(2)M17 clones expressing miR-142 and miR-null.

Gene Symbol	Gene Array		qRT-PCR	
	Fold Change	p value	Fold Change	p value
CYP2S1	−3.79	4.2E-06	N.D.	N.D.
DEPDC6	−4.23	6.3E-05	−1.27	0.318
ADAMTS2	−2.85	6.4E-05	−11.79	0.110
AKAP12	−2.33	1.6E-04	−3.07	0.007
BAMBI	−3.21	3.7E-04	−2.97	0.155
GNG8	−3.09	4.1E-04	−1.19	0.441
MAOA	−2.81	8.3E-04	−5.74	0.013
PLD5	−2.66	9.7E-04	−3.07	0.146

Genes that were found to be down-regulated in the miR-142 clones with a p-value <0.001 in the microarray analysis are shown here. Only MAOA and AKAP12 showed significant (p < 0.05) down-regulation in the post-validation experiment using RT-PCR.

### miR-142-overexpressing clones have lower MAOA protein levels and enzyme activity

**Figure 1:**
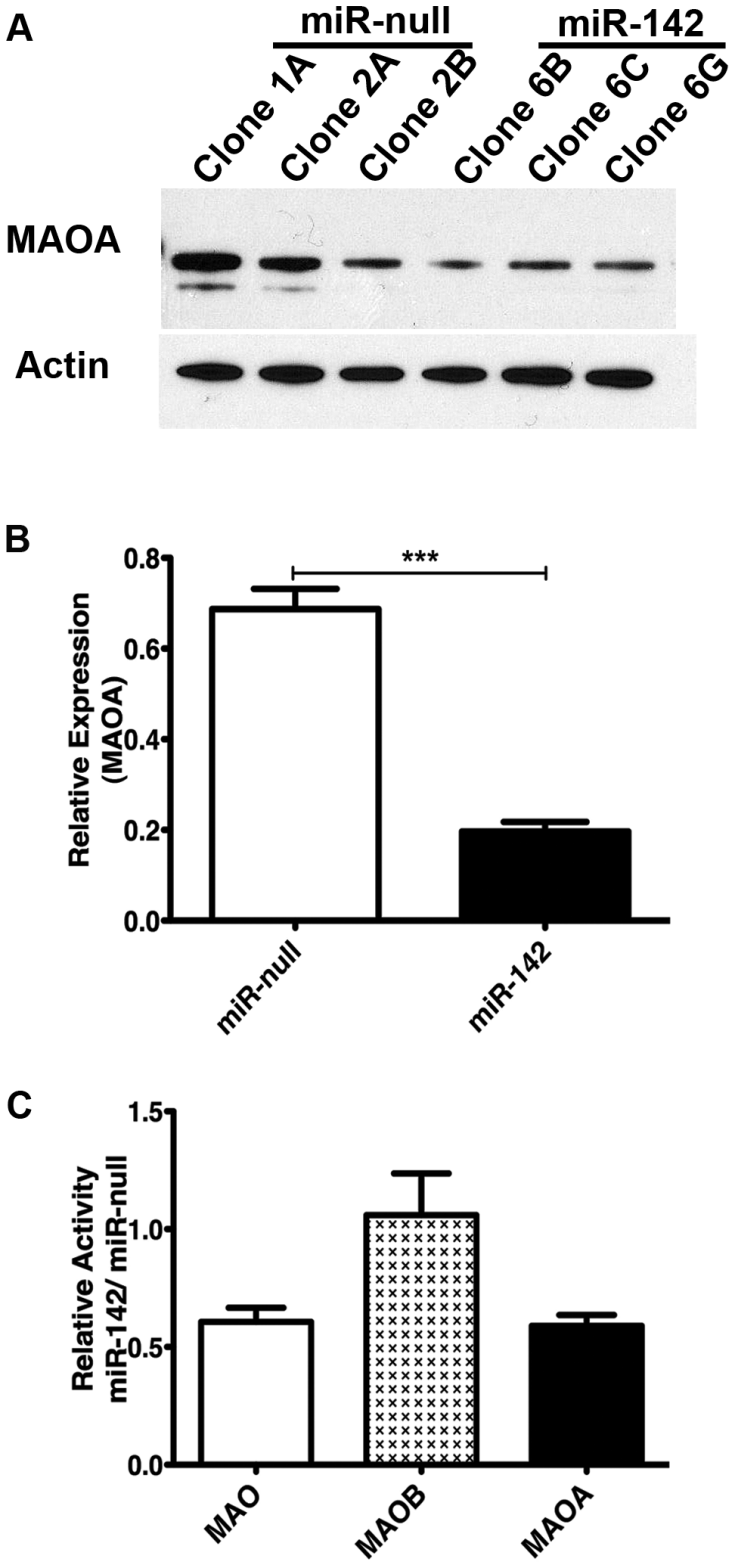
miR-142 downregulates MAOA protein expression and enzyme activity in BE(2)M17 cells. (A) Representative Western blot for MAOA in stable BE(2)M17 clones expressing miR-142 and in control miR-null clones. (B) Quantification of three independent Western blots showing that MAOA protein level is lower (-3.5 fold) in stable BE(2)M17 clones expressing miR-142 compared to miR-null clones. Blots were normalized to β-actin and unpaired t-test was performed. *** represent p<0.001, error bars are standard error of mean. (C) Total MAO as well as MAOA and MAOB specific activity was measured in the miR-142 and miR-null BE(2)M17 clones. Both total MAO and MOA specific activity was approximately 40% lower in miR-142 clones compared to miR-null clones. There was no difference in MAOB specific activity. Y-axis represents fold change of enzyme activity, error bars represent standard error of mean, n = 3.

Western blot analysis was performed with whole cell lysates from three stable BE(2)M17 clones expressing miR-142 (clones 6B, 6C and 6G) and three miR-null clones (1A, 2A, 2B). In addition to reduction in MAOA mRNA level, MAOA protein level was 3.5 fold lower in the miR-142-expressing clones compared to the miR-null clones (Figure 1 A, B).

Next we investigated whether the decrease in MAOA expression in neuronal cells translates to a deficit in its activity. There are two enzymes with MAO activity in cells, MAOA and MAOB, both of which are localized to the outer membrane of the mitochondria. We isolated mitochondria from miR-142 and miR-null clones to enrich for the MAOs, and tested each for MAO enzyme activity, using the specific inhibitors, clorgyline to inhibit MAOA activity and pargyline to inhibit MAOB activity. Similar to the protein levels, both total MAO and MAOA specific activity was found to be about 40% lower in miR-142 clones compared to miR-null clones. There was no difference in MAOB activity (Figure 1C).

### Overexpression of miR-142 in primary human neurons leads to decrease in MAOA protein levels

**Figure 2:**
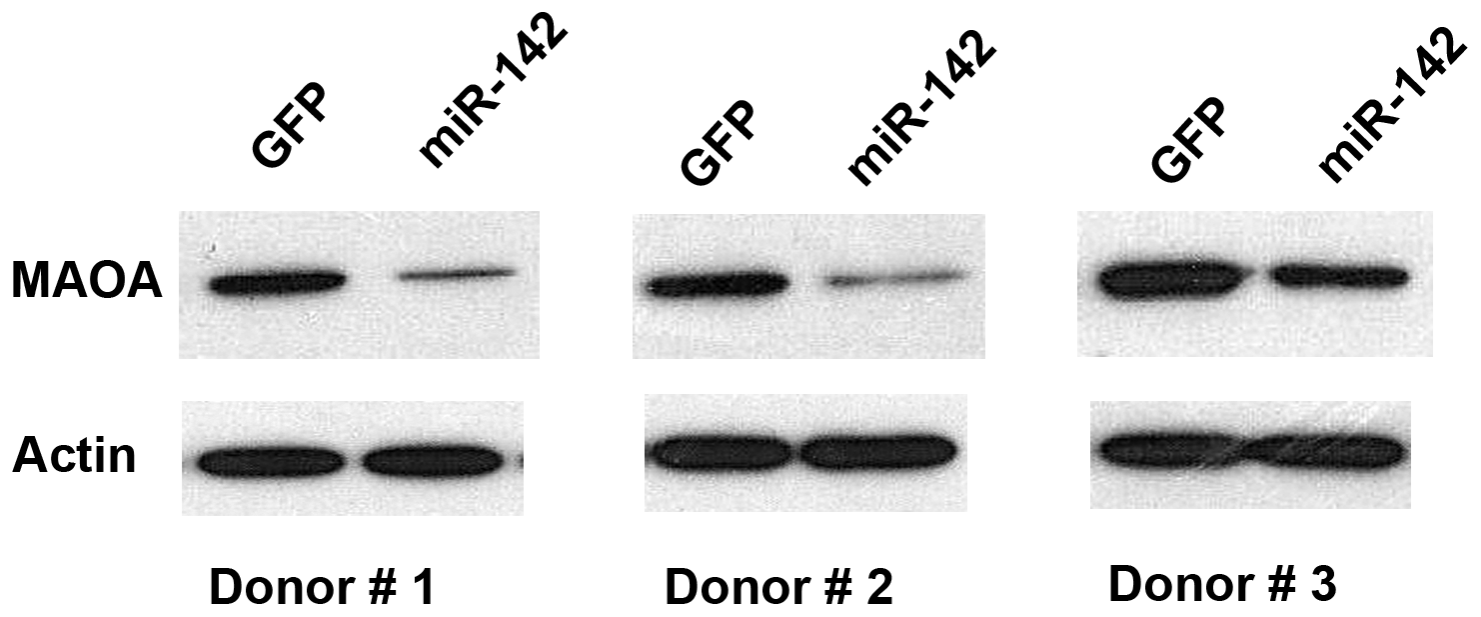
miR-142 downregulates MAOA protein expression in human neurons. Human neurons grown in vitro for 11 days were transduced with either miR-142 or GFP lentivirus. On day 6 after transduction, cells were harvested and Western blot analysis was performed for MAOA. Representative Western blots from three human neuron donors indicating a decrease in MAOA protein level after miR-142 transduction compared to control GFP transduction are shown. Quantification of Western blots from 5 human neuron donors revealed a significant decrease in MAOA level after miR-142 transduction (fold change -1.9, p<.05). Blots were normalized to β-actin and paired t-test was performed.

To confirm that these observed effects also occur in primary neurons, human neurons were cultured *in vitro* for 11 days. They were then transduced with either a miR-142 expressing lentivirus or a control GFP lentivirus. Cells were harvested 6 days after transfection. Expression levels of miR-142-3p and -5p were confirmed by performing qRT-PCR. Western blot for MAOA with whole cell lysates from five independent primary human neuron donors revealed that MAOA protein level was 1.9 fold lower (p<0.05, n = 5) in neurons transduced with miR-142 compared to those transduced with the control GFP lentivirus (Figure 2).

### Overexpression of SIRT1 restores MAOA protein levels in miR-142-overexpressing BE(2)M17 clones

**Figure 3:**
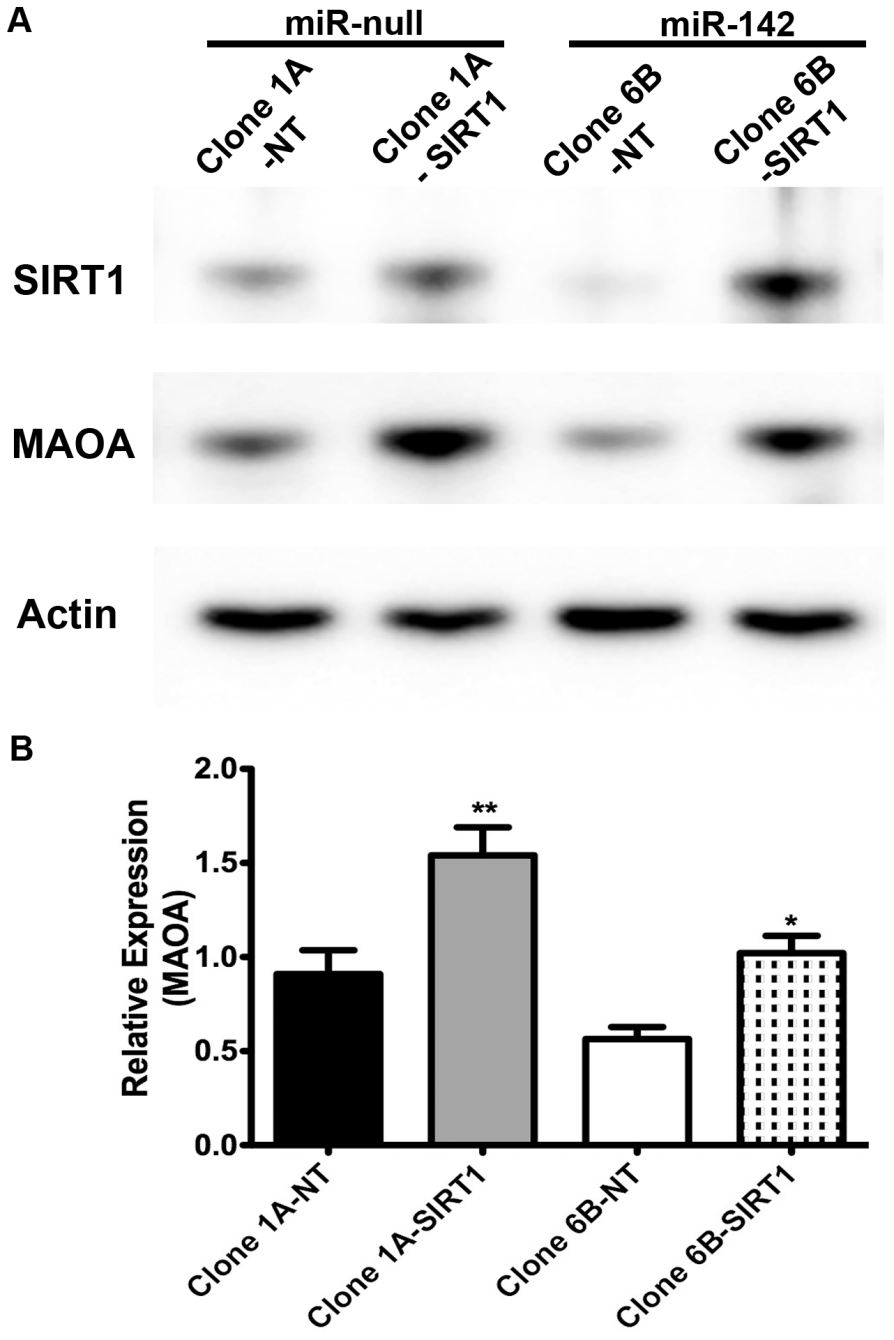
Overexpression of SIRT1 in BE(2)M17 clones leads to increase in MAOA protein levels. (A) Representative Western blot for SIRT1 and MAOA in BE(2)M17 clones 1A (miR-null) and 6B (miR-142). As predicted, clone 6B has lower levels of SIRT1 compared to clone 1A. Transfection of SIRT1 results in overexpression of the same, as well as increase in MAOA protein levels in both the clones. (B) Quantification of MAOA expression by Western blot analysis after three independent transfection experiments. MAOA protein level was increased by 1.7-fold in clone 1A and 1.8-fold in clone 6B after transfection of SIRT1. Blots were normalized to β-actin and one-way ANOVA was performed followed by Bonferroni's multiple correction test. ** represent p<0.01, * represents p<0.05, error bars are standard error of mean, NT =  non-transfected.

Bioinformatic analysis indicated that the MAOA 3′-UTR does not have predicted binding sites for miR-142, and luciferase reporter assays examining revealed no effect of miR-142 on the MAOA 3′-UTR (data not shown). Since miR-142 expression leads to decreased levels of SIRT1 [8], and SIRT1 can act on the MAOA promoter to upregulate its expression [23], we hypothesized that introduction of additional SIRT1 would increase the level of MAOA. We therefore transfected one of the miR-142 (clone 6B) and one of the miR-null clones (clone 1A) with a SIRT1 expression plasmid (lacking the 3′-UTR with the miR-142 recognition site). The cells were harvested 48 hours after transfection. While as expected the Western blot analysis showed that SIRT1 protein level was lower (fold change  = -2.3, p<0.05, n = 3) in the miR-142 expressing clone compared to the miR-null clone, in both clones increased expression of the SIRT1 protein level following the transfections led to an increase in MAOA protein level (miR-null clone 1A, fold change  = 1.7, p<0.01, n = 3; miR-142 clone 6B, fold change  = 1.8, p<0.05, n = 3) (Figure 3A, B).

## Discussion

**Figure 4:**
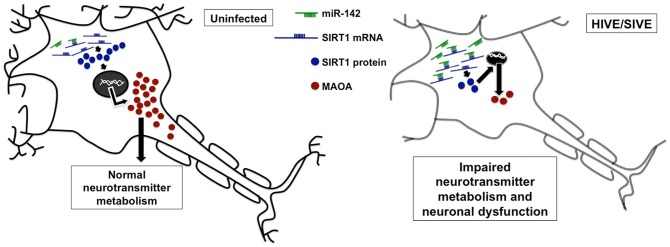
Schematic representation of downstream effects of miR-142 upregulation in neurons. Normally, uninfected neurons have minimal miR-142 expression. Most of the SIRT1 mRNA is therefore available for translation, maintaining the SIRT1 protein pool. SIRT1 deacetylates a lysine residue in the transcription factor NHLH2 and activates it. NHLH2 in turn increases MAOA transcription. In HIVE/SIVE, miR-142 expression is upregulated in neurons. One of the two mature strands of this miR, miR-142-5p, binds to the 3′UTR of SIRT1 mRNA and prevents its translation. This leads to reduction of SIRT1 protein level. Therefore SIRT1 is no longer available for induction if MAOA expression, finally resulting in decrease in MAOA expression and activity.

In this study we have elucidated a novel molecular mechanism of miR-mediated regulation of MAOA expression neuronal cells. Chronic overexpression of miR-142 led to decrease in MAOA mRNA and protein levels, as well as a reduction in the enzyme activity. This regulation of MAOA expression and activity by miR-142 is mediated indirectly through repression of the direct miR-142-5p target, SIRT1, that otherwise induces MAOA expression. Decrease in MAOA expression and activity may contribute to the reported changes in neurotransmitter metabolism in HAND, ultimately leading to neuronal dysfunction (Figure 4).

MiR-142 has two functionally active mature strands, miR-142-3p and -5p. In HIVE/SIVE both strands of miR-142 are upregulated [6], [7]. MiR-142 is known to be expressed in the cells of the hematopoietic lineage [13]. However, in brain sections from rhesus macaques with SIVE, co-localization studies with cell type markers showed that in addition to myeloid cells, miR-142 is expressed both in neurons as well [8]. Upregulation of neuronal miR-142 expression was also reported following peripheral nerve crush [17] and cocaine treatment [18]. In order to investigate the downstream effects of such increase in neuronal miR-142 expression we compared gene expression of stable BE(2)M17 clones expressing miR-142 to clones that were transduced with miR-null. Among the significantly differentially expressed genes, we chose to focus on MAOA for further studies.

MAOs are neurotransmitter-metabolizing enzymes located on the outer mitochondrial membrane. There are two isoforms of MAO, MAOA and MAOB, having about 70% sequence homology but distinct substrate specificities. MAOA preferentially deaminates serotonin, melatonin, epinephrine and norepinephrine, while MAOB has higher affinity for dietary amines like phenethylamine. Both isoforms have similar affinity for dopamine [27]. Congruent with its prime role in neurotransmitter catabolism, pharmacological and genetic evidence has linked MAOA with mood and emotion [28]-[32]. We found that miR-142 over-expression in neuronal cell lines and neurons results in lower MAOA mRNA and protein levels, as well as enzyme activity. These cells will therefore have impaired monoaminergic neurotransmitter catabolism.

Dysfunction of the dopaminergic systems has been reported in HAND [33]–[35]. Dopaminergic neurotransmission is reduced in advanced stages of the disease as evidenced by reduction in dopamine levels in post-mortem brain and CSF samples. This may be a result of progressive loss of dopaminergic neurons in the brain. However, in early stages of the disease, an increase in dopaminergic tone has been observed. Treatment naïve asymptomatic HIV patients have higher levels of dopamine in the CSF due to decrease in dopamine turnover, as evidenced by lower CSF levels of the dopamine metabolite, homovallinic acid [33]. This suggests a possible decrease in activity of dopamine-catabolizing enzymes, such as MAO and Catechol-O-Methyl Transferase, early in the disease. Upregulation of miR-142 may contribute to such changes in dopaminergic neurotransmission in the disease by lowering MAOA expression and activity.

Although miR-142 expressing cells had lower MAOA expression and activity, miR-142 cannot target MAOA directly as there are no miR-142 recognition elements in the MAOA 3′UTR. Therefore regulation of MAOA by miR-142 must be mediated by a direct miR-142 target. In this context, the NAD-dependent deacetylase SIRT1, was reported to regulate transcription of MAOA [23]. SIRT1 deacetylates and activates the transcription factor NHLH2, inducing MAOA expression [23]. SIRT1 is also a validated target for miR-142-5p [8]. Cells that express high levels of miR-142 in SIVE have lower SIRT1 protein levels [8]. Decrease in SIRT1 levels due to upregulation of miR-142 could therefore explain this decrease in MAOA expression and activity. Indeed, we found that overexpression of SIRT1 in the BE(2)M17 clones could increase MAOA protein level.

In addition to neurons, upregulation of miR-142 was also observed in myeloid cells in the SIVE brain [8]. Recent studies have demonstrated that myeloid cells express components of the dopaminergic system including dopamine receptors, dopamine transporters and the dopamine synthesizing enzyme tyrosine hydroxylase [36]–[38]. MAOA is also expressed in monocytes/macrophages where it is strongly induced by the Th2 cytokines IL-4 and IL-13; although its function in these cell types is not clear it is speculated that it serves an immunomodulatory role [39]. Further investigation is therefore required to elucidate the functional effect of upregulation of miR-142 and downregulation of MAOA in microglia/macrophages in SIVE.

In conclusion, we found that chronic increase in neuronal miR-142 expression leads to decrease in MAOA expression and activity. This effect of miR-142 on MAOA expression level is mediated by downregulation of the direct miR-142-5p target SIRT1. Upregulation of miR-142 may therefore contribute to changes in dopaminergic neurotransmission reported in HAND.

## References

[1] Sun AX, Crabtree GR, Yoo AS (2013) MicroRNAs: regulators of neuronal fate. Current Opinion in Cell Biology 25: 215–221. 23374323 10.1016/j.ceb.2012.12.007 PMC3836262

[2] Salta E, De Strooper B (2012) Non-coding RNAs with essential roles in neurodegenerative disorders. The Lancet Neurology 11: 189–200. 22265214 10.1016/S1474-4422(11)70286-1

[3] Zhou L, Pupo GM, Gupta P, Liu B, Tran SL, et al (2012) A parallel genome-wide mRNA and microRNA profiling of the frontal cortex of HIV patients with and without HIV-associated dementia shows the role of axon guidance and downstream pathways in HIV-mediated neurodegeneration. BMC Genomics 13: 677. 23190615 10.1186/1471-2164-13-677 PMC3560210

[4] Pacifici M, Delbue S, Ferrante P, Jeansonne D, Kadri F, et al (2013) Cerebrospinal fluid miRNA profile in HIV-encephalitis. J Cell Physiol 228: 1070–1075. 23042033 10.1002/jcp.24254 PMC3760673

[5] Winkler JM, Chaudhuri AD, Fox HS (2012) Translating the Brain Transcriptome in NeuroAIDS: From Non-human Primates to Humans. J Neuroimmune Pharmacol 7: 372–379. 22367717 10.1007/s11481-012-9344-5 PMC3354039

[6] Yelamanchili SV, Chaudhuri AD, Chen LN, Xiong H, Fox HS (2010) MicroRNA-21 dysregulates the expression of MEF2C in neurons in monkey and human SIV/HIV neurological disease. Cell Death and Dis 1: e77. 10.1038/cddis.2010.56 PMC3002786 21170291

[7] Noorbakhsh F, Ramachandran R, Barsby N, Ellestad KK, LeBlanc A, et al (2010) MicroRNA profiling reveals new aspects of HIV neurodegeneration: caspase-6 regulates astrocyte survival. FASEB J 24: 1799–1812. 20097875 10.1096/fj.09-147819

[8] Chaudhuri AD, Yelamanchili SV, Marcondes MC, Fox HS (2013) Up-regulation of microRNA-142 in simian immunodeficiency virus encephalitis leads to repression of sirtuin1. FASEB J. 10.1096/fj.13-232678 PMC3752547 23752207

[9] Kaul M (2009) HIV-1 associated dementia: update on pathological mechanisms and therapeutic approaches. Curr Opin Neurol 22: 315–320. 19300249 10.1097/WCO.0b013e328329cf3c PMC2779773

[10] Antinori A, Arendt G, Becker JT, Brew BJ, Byrd DA, et al (2007) Updated research nosology for HIV-associated neurocognitive disorders. Neurology 69: 1789–1799. 17914061 10.1212/01.WNL.0000287431.88658.8b PMC4472366

[11] Heaton RK, Franklin DR, Ellis RJ, McCutchan JA, Letendre SL, et al (2011) HIV-associated neurocognitive disorders before and during the era of combination antiretroviral therapy: differences in rates, nature, and predictors. J Neurovirol 17: 3–16. 21174240 10.1007/s13365-010-0006-1 PMC3032197

[12] Gersten M, Alirezaei M, Marcondes MC, Flynn C, Ravasi T, et al (2009) An integrated systems analysis implicates EGR1 downregulation in simian immunodeficiency virus encephalitis-induced neural dysfunction. J Neurosci 29: 12467–12476. 19812322 10.1523/JNEUROSCI.3180-09.2009 PMC2802851

[13] Chen CZ, Li L, Lodish HF, Bartel DP (2004) MicroRNAs modulate hematopoietic lineage differentiation. Science 303: 83–86. 14657504 10.1126/science.1091903

[14] Bissels U, Wild S, Tomiuk S, Hafner M, Scheel H, et al (2011) Combined characterization of microRNA and mRNA profiles delineates early differentiation pathways of CD133+ and CD34+ hematopoietic stem and progenitor cells. Stem Cells 29: 847–857. 21394831 10.1002/stem.627 PMC3116150

[15] Jin HL, Kim JS, Kim YJ, Kim SJ, Broxmeyer HE, et al (2012) Dynamic expression of specific miRNAs during erythroid differentiation of human embryonic stem cells. Mol Cells 34: 177–183. 22767248 10.1007/s10059-012-0090-6 PMC3887816

[16] Nishiyama T, Kaneda R, Ono T, Tohyama S, Hashimoto H, et al (2012) miR-142-3p is essential for hematopoiesis and affects cardiac cell fate in zebrafish. Biochem Biophys Res Commun 425: 755–761. 22884798 10.1016/j.bbrc.2012.07.148

[17] Wu D, Raafat M, Pak E, Hammond S, Murashov AK (2011) MicroRNA machinery responds to peripheral nerve lesion in an injury-regulated pattern. Neuroscience 190: 386–397. 21689732 10.1016/j.neuroscience.2011.06.017 PMC3156291

[18] Eipper-Mains JE, Kiraly DD, Palakodeti D, Mains RE, Eipper BA, et al (2011) microRNA-Seq reveals cocaine-regulated expression of striatal microRNAs. RNA 17: 1529–1543. 21708909 10.1261/rna.2775511 PMC3153976

[19] Tobon KE, Chang D, Kuzhikandathil EV (2012) MicroRNA 142-3p mediates post-transcriptional regulation of D1 dopamine receptor expression. PLoS One 7: e49288. 23152889 10.1371/journal.pone.0049288 PMC3495858

[20] Shende VR, Goldrick MM, Ramani S, Earnest DJ (2011) Expression and rhythmic modulation of circulating microRNAs targeting the clock gene Bmal1 in mice. PLoS One 6: e22586. 21799909 10.1371/journal.pone.0022586 PMC3142187

[21] Tan X, Zhang P, Zhou L, Yin B, Pan H, et al (2012) Clock-controlled mir-142-3p can target its activator, Bmal1. BMC Mol Biol 13: 27. 22958478 10.1186/1471-2199-13-27 PMC3482555

[22] Shende VR, Neuendorff N, Earnest DJ (2013) Role of miR-142-3p in the Post-Transcriptional Regulation of the Clock Gene Bmal1 in the Mouse SCN. PLoS One 8: e65300. 23755214 10.1371/journal.pone.0065300 PMC3673942

[23] Libert S, Pointer K, Bell EL, Das A, Cohen DE, et al (2011) SIRT1 activates MAO-A in the brain to mediate anxiety and exploratory drive. Cell 147: 1459–1472. 22169038 10.1016/j.cell.2011.10.054 PMC3443638

[24] Pfaffl MW (2001) A new mathematical model for relative quantification in real-time RT-PCR. Nucleic Acids Res 29: e45. 11328886 10.1093/nar/29.9.e45 PMC55695

[25] Wang X, McManus M (2009) Lentivirus production. J Vis Exp. 10.3791/1499 PMC2865973 19801965

[26] Brunet A, Sweeney LB, Sturgill JF, Chua KF, Greer PL, et al (2004) Stress-dependent regulation of FOXO transcription factors by the SIRT1 deacetylase. Science 303: 2011–2015. 14976264 10.1126/science.1094637

[27] Naoi M, Maruyama W, Inaba-Hasegawa K, Akao Y (2011) Type A monoamine oxidase regulates life and death of neurons in neurodegeneration and neuroprotection. Int Rev Neurobiol 100: 85–106. 21971004 10.1016/B978-0-12-386467-3.00005-4

[28] Bortolato M, Chen K, Shih JC (2008) Monoamine oxidase inactivation: from pathophysiology to therapeutics. Adv Drug Deliv Rev 60: 1527–1533. 18652859 10.1016/j.addr.2008.06.002 PMC2630537

[29] Alia-Klein N, Goldstein RZ, Kriplani A, Logan J, Tomasi D, et al (2008) Brain monoamine oxidase A activity predicts trait aggression. J Neurosci 28: 5099–5104. 18463263 10.1523/JNEUROSCI.0925-08.2008 PMC2430409

[30] Meyer JH, Ginovart N, Boovariwala A, Sagrati S, Hussey D, et al (2006) Elevated monoamine oxidase a levels in the brain: an explanation for the monoamine imbalance of major depression. Arch Gen Psychiatry 63: 1209–1216. 17088501 10.1001/archpsyc.63.11.1209

[31] Schulze TG, Muller DJ, Krauss H, Scherk H, Ohlraun S, et al (2000) Association between a functional polymorphism in the monoamine oxidase A gene promoter and major depressive disorder. Am J Med Genet 96: 801–803. 11121185

[32] Yu YW, Tsai SJ, Hong CJ, Chen TJ, Chen MC, et al (2005) Association study of a monoamine oxidase a gene promoter polymorphism with major depressive disorder and antidepressant response. Neuropsychopharmacology 30: 1719–1723. 15956990 10.1038/sj.npp.1300785

[33] Scheller C, Arendt G, Nolting T, Antke C, Sopper S, et al (2010) Increased dopaminergic neurotransmission in therapy-naive asymptomatic HIV patients is not associated with adaptive changes at the dopaminergic synapses. J Neural Transm 117: 699–705. 20454983 10.1007/s00702-010-0415-6

[34] Kumar AM, Fernandez JB, Singer EJ, Commins D, Waldrop-Valverde D, et al (2009) Human immunodeficiency virus type 1 in the central nervous system leads to decreased dopamine in different regions of postmortem human brains. J Neurovirol 15: 257–274. 19499455 10.1080/13550280902973952 PMC9618304

[35] Gelman BB, Spencer JA, Holzer CE 3rd, Soukup VM (2006) Abnormal striatal dopaminergic synapses in National NeuroAIDS Tissue Consortium subjects with HIV encephalitis. J Neuroimmune Pharmacol 1: 410–420. 18040813 10.1007/s11481-006-9030-6

[36] Gaskill PJ, Carvallo L, Eugenin EA, Berman JW (2012) Characterization and function of the human macrophage dopaminergic system: implications for CNS disease and drug abuse. J Neuroinflammation 9: 203. 22901451 10.1186/1742-2094-9-203 PMC3488577

[37] Gaskill PJ, Calderon TM, Luers AJ, Eugenin EA, Javitch JA, et al (2009) Human immunodeficiency virus (HIV) infection of human macrophages is increased by dopamine: a bridge between HIV-associated neurologic disorders and drug abuse. Am J Pathol 175: 1148–1159. 19661443 10.2353/ajpath.2009.081067 PMC2731133

[38] Gaskill PJ, Calderon TM, Coley JS, Berman JW (2013) Drug induced increases in CNS dopamine alter monocyte, macrophage and T cell functions: implications for HAND. J Neuroimmune Pharmacol 8: 621–642. 23456305 10.1007/s11481-013-9443-y PMC4303241

[39] Chaitidis P, Billett EE, O'Donnell VB, Fajardo AB, Fitzgerald J, et al (2004) Th2 response of human peripheral monocytes involves isoform-specific induction of monoamine oxidase-A. J Immunol 173: 4821–4827. 15470022 10.4049/jimmunol.173.8.4821

